# Spontaneous Separation of the Anterior Portion of the Inferior Maxilary Bone

**Published:** 1844-09

**Authors:** 

**Affiliations:** Pavia.


*Spontaneous Separation of the Anterior Portion of the Inferior Maxillary Bone—by Dr. Gambini, of Pavia.*—Castagnoli Maria, twenty-five years of age, entered the Civil Hospital on the 20th of September, for extensive caries of the inferior maxillary bone, from a scorbutic affection which had existed a long time. A large swelling in the course of this bone was observed; the gums were swollen, fungous, ulcerated, and bled on the least touch; the teeth which remained were either loose or carious; several alveoli were exposed, and a fœtid sanies discharged from the mouth. The patient presented all the characters of a general scorbutic affection. The process of throwing off the bone was left to nature, and the treatment consisted in the administration of the anti-scorbutic wine of Nidman. The use of a simple gargle of barley water and *mel. rosar.* and a vegetable diet were ordered. Several teeth were extracted to facilitate the escape of movable scales of bone, which was followed by a sensible amelioration. It was observed for a long time, that a large portion of the alveolar arch, which was entirely laid bare, became more and more movable, and its separation was hastened by moderate tractions made with the forceps. Lastly; on the 26th of November, the extraction was effected without much resistance or great pain, when it was discovered that the whole of the anterior portion of the lower jaw had been taken away in this portion of bone, which was verified by a careful examination of the bone. The hæmorrhage attending this extraction was not great; and cold water was sufficient to stop it. The anti-scorbutic wine was omitted for some days, because it irritated the interior of the mouth, and a common gargle was substituted. At the end of five days the patient was considerably improved; the gums were entirely cicatrised, and did not present any trace of fungous growth. On the 12th of December, the patient was discharged quite cured.—*Forceps.*

